# Development and validation of prognostic machine learning models for short- and long-term mortality among acutely admitted patients based on blood tests

**DOI:** 10.1038/s41598-024-56638-6

**Published:** 2024-03-11

**Authors:** Baker Nawfal Jawad, Shakir Maytham Shaker, Izzet Altintas, Jesper Eugen-Olsen, Jan O. Nehlin, Ove Andersen, Thomas Kallemose

**Affiliations:** 1 Department of Clinical Research, Copenhagen University Hospital Amager and Hvidovre, Hvidovre, Denmark; 2 Emergency Department, Copenhagen University Hospital Amager and Hvidovre, Hvidovre, Denmark; 3 Department of Clinical Medicine, University of Copenhagen, Copenhagen, Denmark; 4 Department of Data Science, IT University of Copenhagen, Copenhagen, Denmark

**Keywords:** Predictive markers, Prognostic markers, Computer science

## Abstract

Several scores predicting mortality at the emergency department have been developed. However, all with shortcomings either simple and applicable in a clinical setting, with poor performance, or advanced, with high performance, but clinically difficult to implement. This study aimed to explore if machine learning algorithms could predict all-cause short- and long-term mortality based on the routine blood test collected at admission. Methods: We analyzed data from a retrospective cohort study, including patients > 18 years admitted to the Emergency Department (ED) of Copenhagen University Hospital Hvidovre, Denmark between November 2013 and March 2017. The primary outcomes were 3-, 10-, 30-, and 365-day mortality after admission. PyCaret, an automated machine learning library, was used to evaluate the predictive performance of fifteen machine learning algorithms using the area under the receiver operating characteristic curve (AUC). Results: Data from 48,841 admissions were analyzed, of these 34,190 (70%) were randomly divided into training data, and 14,651 (30%) were in test data. Eight machine learning algorithms achieved very good to excellent results of AUC on test data in a of range 0.85–0.93. In prediction of short-term mortality, lactate dehydrogenase (LDH), leukocyte counts and differentials, Blood urea nitrogen (BUN) and mean corpuscular hemoglobin concentration (MCHC) were the best predictors, whereas prediction of long-term mortality was favored by age, LDH, soluble urokinase plasminogen activator receptor (suPAR), albumin, and blood urea nitrogen (BUN). Conclusion: The findings suggest that measures of biomarkers taken from one blood sample during admission to the ED can identify patients at high risk of short-and long-term mortality following emergency admissions.

## Introduction

Prognostic tools predicting all-cause mortality are crucial for decision making in Emergency Departments and Intensive Care Units (ICU). Tools predicting disease severity and mortality have been inquired for effective patient management and resource allocation to ensure appropriate treatment and evaluate medications, protocols, and interventions^1^. Consequently, various scores and indices have been proposed to predict mortality, such as Acute Physiologic Assessment and Chronic Health Evaluation (APACHE)^2^, National Early Warning Score (NEWS)^3,4^, Modified Early Warning Score (MEWS)^5^, Mortality Probability Models^6^, Sequential Organ Failure Assessment (SOFA)^7^, Emergency Severity Index (ESI)^8^, and Cardiac Arrest Risk Triage score (CART)^9^. Lately, Geriatric scores have also been proposed, such as the Barthel Index^10,11^, the Clinical Frailty Score^12^, and FI-OutRef, a frailty index, calculated as the number of admission laboratory test results outside of the reference interval based upon blood collected at the admission time in + 65 years old acutely admitted patients^13^. The majority of the existing score systems are based on a specifically defined patient cohort and target specific conditions. Furthermore, with an area under the curve (AUC) of 0.68–80^14,15^, these scores have only moderate accuracy in predicting short-term mortality, and are not developed for predicting long-term mortality. The existing scores are typically based on physiological and laboratory parameters based on a simple linear relationship. However, considering the global aging phenomenon and the increase in the prevalence of multimorbidity and polypharmacy, the proportion of complex patients has increased^16–20^. As a result, these scores are simple and cannot elucidate the complexity, and the clinical requirements for use in daily clinical practice are not met^21–23^. In recent years, many studies have shown the significant potential of applying advanced machine learning (ML) algorithms in healthcare data^24–27^. Several ML algorithms have been explored in healthcare to assist with diagnosis and prognosis, including the prediction of short and long-time mortality^28–35^. For instance, 30-day and up to 4-year mortality risk models have been explored for medical and surgical patients discharged from the hospital with ROC-AUC of 0.95–0.96^36,37^, and with a balanced-accuracy between 65 and 67% for 4 year-mortality^38^. For in-hospital mortality prediction, Li et al. (2021) achieved an excellent AUC of 0.97 using ML algorithms based on 76 combined invasive and non-invasive parameters^39^. For 30-day mortality risk after discharge, Blom et al.^36^ achieved an excellent discrimination AUC of 0.95, using data from electronic health records, morbidity scores, information about the referred doctor, ambulance transport, previous emergency medical condition, information about radiological order, discharge time and days in the hospital, and triage priority. However, to our knowledge, most existing models use various parameters, such as demographics, patient history, morbidity, medication, and non-invasive and invasive parameters, to predict mortality, which is difficult for clinicians to interpret and implement in a flow culture setting such as the ED^40^. Furthermore, very few studies have investigated ML modeling for all-cause short- and long-term mortality risk in a general population cohort at the ED. Hence, the aim of this study was to explore, develop and validate ML algorithms which can predict all-cause short—and long-term mortality based on few or easily measured routine blood samples collected at admittance at the emergency department.

## Methods

### Study design and settings

In this study, we analyzed data from a retrospective cohort study from the Emergency Department at the Copenhagen University Hospital, Amager and Hvidovre. The cohort included all patients admitted to the Acute Medical Unit of the Emergency Department with an available blood sample during the follow-up between 18 November 2013 and 17 March 2017, whose follow-up data are available in the Danish National Patient Registry (DNPR). The Acute Medical Unit receives patients within all specialties, except children, gastroenterological patients, and obstetric patients. The follow-up period began from admission and extending to 90 days after discharge for the last patient was included, corresponding to a median follow-up time of 2 years: a range of 90–1.301 days. During the study period, patients who left the country for an extended length of time were censored at the time they were last admitted. This study was reported in accordance with the Transparent reporting of a multivariable prediction model for individual prognosis or diagnosis (TRIPOD): The TRIPOD statement^41^.

### Biomarkers

On admission, blood samples were taken, and a standard panel of markers was measured at the Department of Clinical Biochemistry, including C-reactive protein (CRP), Soluble urokinase plasminogen activator receptor (suPAR), Alanine Aminotransferase (ALAT), Albumin (ALB), International Normalized Ratio (INR), coagulation factors 2,7,10 (KF2710), total Bilirubin (BILI), Alkaline Phosphatase, Creatinine, Lactate dehydrogenase (LDH), Blood urea nitrogen (BUN), Potassium (K), Sodium (NA), Estimated Glomerular Filtration Rate (eGFR), Hemoglobin (HB), mean corpuscular volume (MCV) and mean corpuscular hemoglobin concentration (MCHC), number of leukocytes, lymphocytes, neutrocytes, monocytes, thrombocytes, eosinophils, basophils, and Metamyelo-, Myelo.—Promyelocytes (PROMM)^42^. Age and sex were also included as variables in the algorithms (Table 1).

**Table 1:** Baseline characteristics of patients’ blood test results. Stratified by time of death within, 3, 10, 30, and 365 days after admission at the emergency department.

	**Total**	**Survival**	**3-day mortality**	**10-day mortality**	**30-day mortality**	**365-day mortality**
Number of patients	28,671	23,039				
Mortality rate (%)	5632 (19.6%)	0	201 (0.7%)	1252 (4.4%)	2338 (8.2%)	4677 (16.3%)
Variables						
Age	65.6 (48.2: 78.5)	54.7(38.0: 70.0)	80.6 (71.5: 87)	80.8 (71.7: 88.2)	80.4 (71: 87.7)	79.1 (69.5: 86.5)
Sex (female)	15,175 (52.9%)	1231 (53.4%)	184 (51.8%)	668 (52%)	1376 (51.5%)	4224 (49%)
ALAT (U/L)	21 (15: 33)	22 (16: 33)	31 (19: 82)	25 (16: 52)	22 (15: 41)	19 (13: 32)
Albumin (g/L)	34 (30: 37)	36(33: 36)	27 (22: 31)	27 (22: 31)	27 (22: 31)	29 (25: 33)
Basophils (× 10^9 /L)	0.03 (0.02: 0.05)	0.03(0.02: 0.05)	0.03 (0.02: 0.05)	0.03 (0.01: 0.05)	0.03 (0.01: 0.05)	0.03 (0.02: 0.05)
Alkaline Phosphatase (U/L)	76 (63: 94)	69 (56: 85.8)	123 (90: 176)	114 (83: 152)	114 (82: 149)	98 (75: 129)
Bilirubin (µmol/L)	7 (5: 10)	7 (6: 10)	9 (6: 17)	10 (6: 16)	9 (6: 14)	8 (5: 13)
BUN (mmol/L)	5.1 (3.8: 7.2)	4.7 (3.5: 6)	11.5 (7.1: 19.5)	10.8 (6.7: 17.4)	9.6 (6.1: 15.8)	7.6 (5.1: 12.3)
Creatinine (µmol/L)	77 (62: 97)	74 (62:89)	123 (82: 190)	107 (72: 171)	98 (67: 153)	90 (66: 132)
CRP (mg/L)	7 (2: 39)	4 (1:19)	76 (20.5: 180)	73 (27: 160)	67 (22: 148.5)	38 (9: 95)
HB (mmol/L)	8.1 (7.2: 8.9)	8.4 (7.7–9.1)	7.3 (6.3: 8.5)	7.2 (6.2: 8.3)	7.1 (6.2: 8.1)	7.2 (6.3: 8.1)
INR	1 (1: 1.1)	1 (1: 1.1)	1.2 (1: 1.4)	1.1 (1: 1.3)	1.1 (1: 1.3)	1.1 (1: 1.2)
Potassium (mmol/L)	3.9 (3.6: 4.2)	3.8 (3.6: 4.1)	4.3 (3.8: 5.1)	4.1 (3.6: 4.6)	4 (3.6: 4.5)	4 (3.6: 4.3)
KF2710	0.91 (0.77: 1.02)	0.92 (0.77: 1.01)	0.74 (0.55:0.89)	0.76 (0.56: 0.91)	0.77 (0.58: 0.91)	0.83 (0.66: 0.96)
LDH (U/L)	186 (169: 214)	175 (151:205)	334 (267: 410)	289 (229: 355)	268 (219: 328)	224(188: 274)
Leukocytes (× 10^9 /L)	8.7 (6.9: 11.3)	8.5 (6.9: 10.8)	13.7 (9.6: 19.6)	12.7 (9.1: 17.0)	11.8 (8.6: 16.0)	10.1 (7.6: 13.8)
Lymphocytes (× 10^9 /L)	1.7 (1.1: 2.3)	1.8 (1.3: 2.5)	1.2 (0.7: 1.9)	1 (0.6: 1.6)	1.1 (0.7: 1.6)	1.2 (0.8: 1.8)
MCHC (mmol/L)	20.7 (20.1: 21.2)	20.9 (20.4: 21.4)	19.8 (19.1:20.5)	20 (19.3: 20.6)	20 (19.4: 20.7)	20.2 (19.5: 20.8)
MCV (fL)	89 (86: 93)	88 (85: 92)	93 (88: 98)	92 (87: 97)	91 (87: 96)	91 (87: 95)
Monocytes (× 10^9 /L)	0.7 (0.5: 0.9)	0.6 (0.5: 0.8)	0.8 (0.5: 1.3)	0.8 (0.5: 1.16)	0.8 (0.51: 1.1)	0.8 (0.53: 1.02)
Neutrocytes (× 10^9 /L)	5.8 (4.1: 8.3)	5.4 (4.: 7.6)	10.9 (7.4: 15.4)	10.3 (6.9: 14.2)	9.5 (6.4: 13.4)	7.6 (5.3: 11.2)
Promm (× 10^9 /L)	0.03 (0.02: 0.06)	0.03 (0.02: 0.05)	0.11 (0.05: 0.29)	0.09 (0.04: 0.19)	0.08 (0.04: 0.16)	0.05 (0.03: 0.11)
suPAR (ng/ml)	3.3 (2.3: 5.0)	2.6 (1.9–3.6)	7.3 (5.2: 10.8)	7.0 (4.9: 10.2)	6.7 (4.7: 9.7)	5.7 (4.0: 8.2)
Thrombocytes (× 10^9/L)	247 (201: 302)	242 (201: 292)	266 (196: 350)	259 (193: 350)	266 (200: 354)	260 (199: 338)
Eosinophils (× 10^9 /L)	0.11 (0.04: 0.19)	0.11 (0.04: 0.21)	0.01 (0: 0.05)	0.01 (0: 0.07)	0.028 (0: 0.10)	0.07 (0.01: 0.17)
eGFR (mL/min)	80 (60: 90)	87 (70: 90)	42 (25: 69)	51 (29: 77)	56 (34: 83)	62 (40: 86)
Sodium (mmol/L)	139 (136: 141)	139 (137: 141)	138 (134: 142)	138 (134: 142)	138 (134: 142)	138 (135: 141)

Baseline results from last admission. Results are expressed as median (IQR interquartile range) for continuous variables. For categorical variables, results are expressed as number of participants (percentage). ALAT, Alanine-aminotrasferase; BUN, blood urea nitrogen; CRP, C-reactive protein; HB, Hemoglobin; INR, prothrombin time and international normalized ratio; KF2710, coagulation factors 2,7,10; LDH, lactate dehydrogenase; MCV, mean corpuscular volume; MCHC, mean corpuscular hemoglobin concentration; Promm, Metamyelo-, Myelo. – Promyelocytes; suPAR, soluble urokinase plasminogen activator receptor; eGFR, estimated glomerular filtration rate.

From The Danish Civil Registration System demographic information, including age, sex, and death time was collected. This study adhered to regional and national regulatory standards, receiving approvals from relevant Danish authorities. Permissions were granted by the Danish Data Protection Agency (ref. HVH-2014-018, 02767) ensuring adherence to data protection regulations, the Danish Health and Medicines Authority (ref. 3-3013-1061/1) for compliance with health and medical standards, and The Capital Region of Denmark, Team for Journaldata (ref. R-22041261), for the use and management of healthcare data within the region. These approvals collectively ensured that the study met the ethical and legal requirements pertaining to research in Denmark.

### Outcomes

In this study, the primary outcomes were 3-,10-,30-, and 365-day mortality, defined as deaths within 3, 10, 30, and 365 days after admission at the emergency department, resulting in binary outcomes (0 = survive, 1 = dead).

### Statistical analysis

R version (4.1.0) and Python (version 3.8.0) was used for statistical analysis in the demographic statistics part of this study. Categorical variables were described as numbers and percentages (%) and continuous variables were described as medians with interquartile range (IQR) for the groups.

### Data preparation

First the data format was unified. Secondly, we excluded patient admissions from the analysis if more than 50% of their clinical biochemistry results were missing. For the admissions included in the study, the median percentage of missing variables was 2.6%, with an interquartile range (IQR) from 2.6 to 7.7%. For missing values, iterative imputations were used from scikit-learn package^43^. For the unequal distribution of our target outcome (imbalance data), several resampling methods were explored, including the random undersampling, the random oversampling, and SMOTE^44,45^.

In this study, we used the random oversampling from imbalanced-learn package^46^ to handle the imbalanced classification distribution best. Outliers were removed using an Isolation Forest. The default setting is 0.05, resulting as 0.025 of the values on each side of the distribution's tail were dropped from the training set. To reduce the impact of magnitude in the variance, we normalized the values of all variables in the data by z-score. To make all variables more normal-distributed like, we power transformed the data by the Yeo-Johnson method^47^.

### Model construction

In this study we used the PyCaret's classification module to train fifteen different algorithms, resulting in a total of 480 models for the four outcomes with a set of 27, 20, 15, 10, 5, 3, 2, 1 biomarker(s). PyCaret (version 2.2.6)^48^, is an automated machine learning low-code library in Python that automates the ML workflow. For all models Python (version 3.8.0) were used. By default, the random selection method was used to split the data into training and test sets of 70% and 30%, respectively. For hyperparameter tuning, a random grid search was used in PyCaret. There was no significant difference between training and test sets after split considering variable values.

### Algorithm selection and performance measures

The fifteen machine learning algorithms (Random Forest (RF), SVM-Radial Kernel (RBFSVM), Extra Trees Classifier (ET), Extreme Gradient Boosting (XGBOOST), Decision Tree Classifier (DT), neural network (MLP), Light Gradient Boosting Machine(LIGHTBM), K Neighbors Classifier (KNN), Gradient Boosting Classifier (GBC), CatBoost Classifier (CATBOOST), Ada Boost Classifier (ADA), Logistic Regression (LR), Linear Discriminant Analysis (LDA), Quadratic Discriminant Analysis (QDA) and Naive Bayes(NB)), were trained and evaluated first on tenfold cross-validation, then on test data. Model selection was based on the Area under the receiver operating characteristic curve (AUC) measure. Additionally, sensitivity, specificity, positive predictive value, and negative predictive value for the complete data, based on probability threshold of 0.5, were estimated for the training and test data and evaluated between them. Similarly, analyses were performed on the top ML models, including a sensitivity analysis using data without variable imputation, and models that included both routine biomarkers and co-morbidity based on ICD-10 codes as features. Out of 4400 possible diagnoses, we selected 389 codes based on a prevalence over 50, focusing on those with a prevalence where at least 50 patients have the diagnosis (Supplementary table 3 and 4).

### Biomarker selection

In this study we aimed to use few biomarkers for predicting mortality. This can reduce the risk of over-fitting, improve accuracy, and reduce the training time^49^. Biomarker selection (Feature Selection) was achieved in PyCaret using various permutation importance techniques depending on the type of model being evaluated. These included Random Forest, Adaboost, and linear correlation with the mortality outcome to select the subset of the most relevant biomarkers for modeling. By default, the threshold used for feature selection was 0.8^50^. During iteration, all biomarkers were fed into each of the models, the best biomarkers were kept, and seven to one biomarker were removed, resulting in models starting with 27 variables and decreasing to 1.

## Results

**Figure 1:**
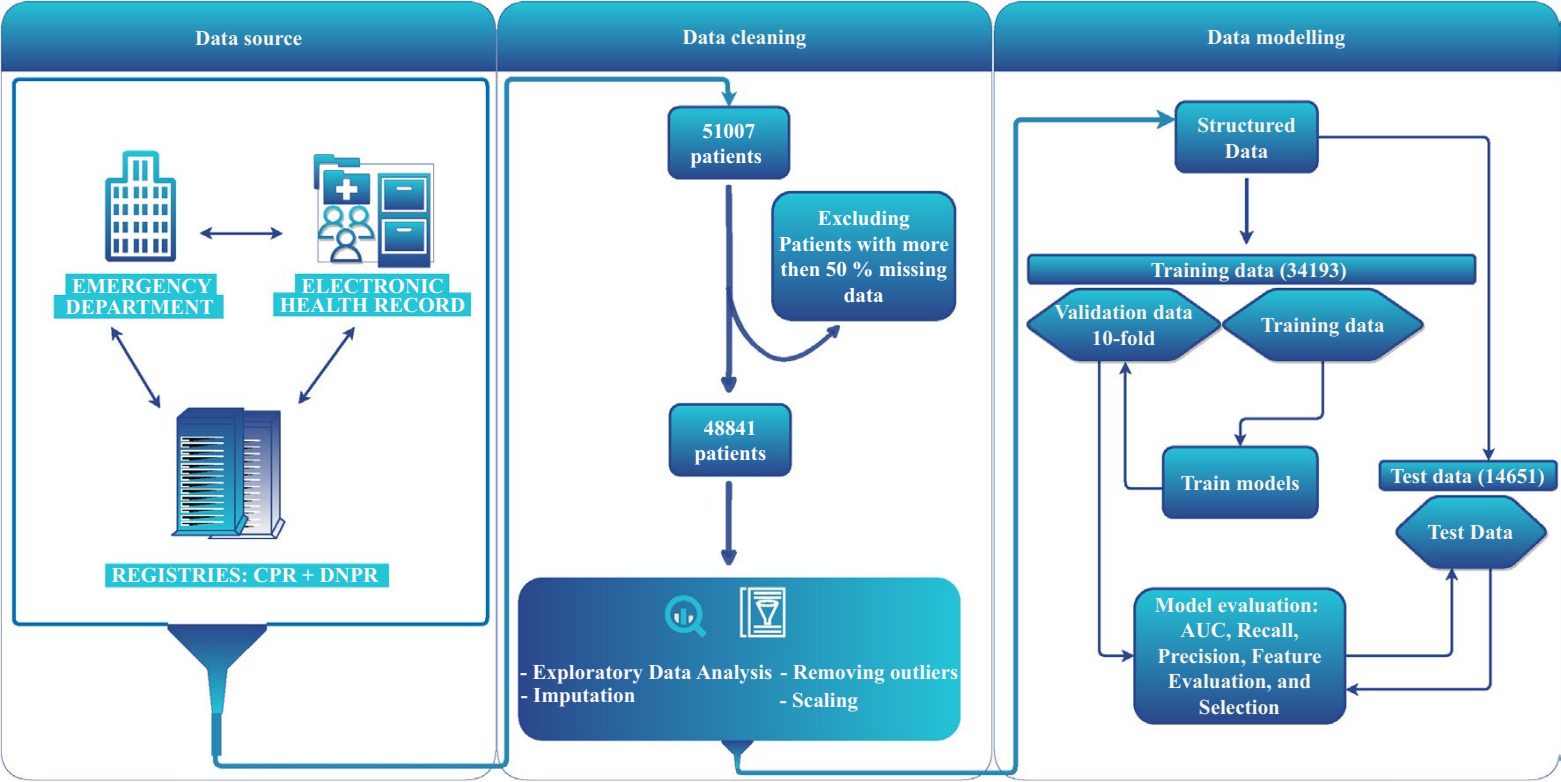
Flowchart of data. We used a cohort study of 51,007 acute patient admissions at the emergency department with laboratory and demographical data. At data pre-processing we excluded 2166 records with more the 50% missing in data (4.3% of the total), removed outliers, imputed and scaled the data. In total 48,841 records were structured data, where 34,190 (70%) patient records were allocated to training data and 14,651 (30%) patient records were allocated to validation and test data.

Figure 1 shows the flow of data. Between 18 November 2013 and 17 March 2017, a total of 51,007 ED admissions occurred during this period. Of these 2166 patient records were excluded due to missing data on more than 50% of variables, resulting in a study cohort of 48,841 admissions obtained from 28,671 unique patients. Randomly, 34,193 (70%) patient records were allocated to training data and 14,651 (30%) patient records were allocated to test data. Table 1 shows the baseline characteristics of patients, at latest admission, median age was 65.6 (IQR: 48.2 – 78.5) years and 52.3% were female. A total of 5632 (19.6%) patients did not survive during the follow-up (see Methods). The differences between not-survived patient at different times follow-up, are shown in Table 1. The mortality rates were 0.7%, 2.4%, 4.6% and 12.7% at 3-day, 10-day, 30-day and 365-day follow-up, respectively. Patients excluded due to missing data showed no significant differences compared to those retained in the study. Additionally, we conducted a sensitivity analysis fitting the top models with and without the imputed data, and demonstrated almost comparable outcomes (except NB and QDA models), indicating that there was no notable impact of imputation on our results (Table S4).

### Model performance

**Figure 2:**
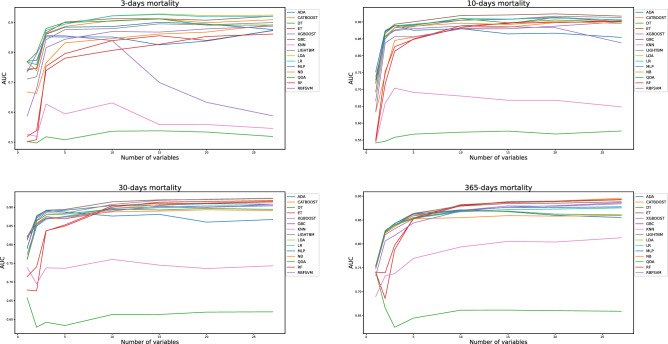
Predictive performance of fifteen ML algorithms on training data, as measured by the Area Under the Receiver Operating Characteristic Curve (AUC) when using 1 to 27 variables as predictors in the machine learning algorithms. Figure 2a–d demonstrate the predictive performance for 3-day, 10-day, 30-day and 365-days mortality, respectively. Among all models, the highest predictive performances in AUC are shown between 0.90 and 0.93 in Figure 2a–d. When using five variables, the top 3 models achieved an AUC of 0.89 in Fig. 2a–c, and an AUC of 0.86 or above when using five variables in Fig. 2d. The AUC falls below 0.85 when using fewer than three variables for all models in Fig. 2a–d.

**Figure 3:**
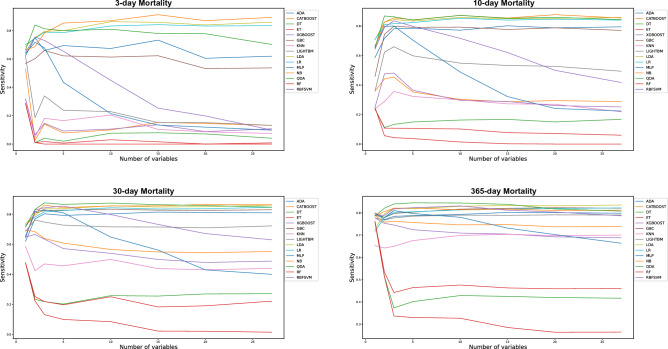
Sensitivity of fifteen ML algorithms on training data, as measured by the Area Under the Receiver Operating Characteristic Curve (AUC) when using 1 to 27 variables as predictors in the machine learning algorithms. Figure 3a–d demonstrate the sensitivity for 3-day, 10-day, 30-day and 365-day mortality on training data, respectively. Among all models, the highest sensitivity is shown between 0.88 and 0.91 in Fig. 3a–c. In Fig. 3d, the highest sensitivity reached is 0.85. When using ten variables, the top 3 models achieved a sensitivity above 0.85–91 in Fig. 3a–d, The Sensitivity falls below 0.8 when using fewer than three variables for all models in Fig. 3a–d.

In Figs. 2a–d and 3a–d, the performance, as denoted by AUC and sensitivity of all fifteen ML models are shown (models described in methods section), respectively. The datasets used in the models included all 26 biomarkers from the routine blood tests and sex as an additional variable. Feature selection (see method section) ranked the most important biomarkers, removing seven to one variable in every iteration, resulting in models ranging from 27 to 1 variable. Based on test data, the AUC of all models ranged between 0.5 and 0.93 and the sensitivity ranged between 0.00 and 0.91 (Fig. 2). Eight of the fifteen models achieved very good to excellent results on test data with an AUC of 0.85–0.93 with a sensitivity > 0.80 using more ten variables (Fig. 2, and supplementary Table 2). Six of the ML algorithms, the Gradient Boosting Classifier (GBC), Light Gradient Boosting Machine (LightGBM), Linear Discriminant Analysis (LDA), Logistic regression (LR), Naïve Bayes (NB) and Quadratic Discriminant Analysis (QDA) had particularly high AUCs > 0.85 and sensitivity > 0.80, even when using only ten variables (Fig. 2). After reducing the number of variables to five, the performance in AUC showed very good performance and high sensitivity > 0.80 in two specific ML models the Gradient Boosting Classifier and the Quadratic Discriminant Analysis (Fig. 2a–c). The Gradient Boosting Classifier achieved an AUC of 0.89 for prediction of 3-day, 10-day, and 30-day mortality, with a sensitivity of 0.85, 0.83, and 0.83, respectively. For prediction of 365-day mortality, the ML algorithm Quadratic Discriminant Analysis had the highest AUC of 0.86, with a sensitivity of 0.80 (Fig. 2d). Using fewer than five variables resulted in a significant decrease in all models to below 0.85 in AUC and below 0,80 in sensitivity (Fig. 2a–d). Further performance metrics for all models can be found in supplementary (Table 2).

**Table 2:** Results from test data for top 3 models predicting short- and long-term mortality.

	**AUC**	**Sensitivity**	**Specificity**	**PPV**	**NPV**	**Number of variables**
3-day Mortality						
Naive bayes	0.91 [0.91—0.91]	0.92 [0.91—0.92]	0.78 [0.78—0.79]	0.03 [0.3–0.3]	0.99 [0.99—0.99]	15
Linear discriminant analysis	0.93 [0.93—0.93]	0.89 [0.86—0.89]	0.83 [0.82—0.83]	0.04 [0.4–0.5]	0.99 [0.99—0.99]	15
Logistic regression	0.93 [0.93—0.93]	0.85 [0.83—0.86]	0.85 [0.84—0.89]	0.04 [0.4–0.4]	0.99 [0.99—0.99]	15
10-day mortality						
Linear discriminant analysis	0.91 [0.90—0.91]	0.90 [0.87—0.93]	0.78 [0.78—0.79]	0.1 [0.09—0.11]	0.99 [0.99—0.99]	10
Logistic regression	0.91 [0.89—0.93]	0.90 [0.87—0.93]	0.79 [0.79—0.79]	0.1 [0.09—0.11]	0.99 [0.99—0.99]	10
Quadratic discriminant analysis	0.90 [0.90 –0.90]	0.91 [0.87—0.93]	0.77 [0.76—0.77]	0.1 [0.08—0.10]	0.99 [0.99—0.99]	10
30-day Mortality						
Linear discriminant analysis	0.90 [090—0.90]	0.90 [0.87—0.92]	0.78 [0.77- 0.79]	0.19 [0.18–0.21]	0.99 [0.99—0.99]	10
Quadratic discriminant analysis	0.91 [0.89—0.91]	0.89 [0.86—0.91]	0.76 [0.75—077]	0.18 [0.17—0.19]	0.99 [0.99—0.99]	10
Gradient boosting classifier	0.92 [0.92—0.92]	0.86 [0.84—0.89]	0.82 [0.82—0.83]	0.22 [0.21—0.24]	0.99 [0.99—0.99]	10
365-day mortality						
Gradient boosting classifier	0.88 [0.88—0.89]	0.82 [0.81—0.83]	0.77 [0.76—0.77]	0.44 [0.43—0.46]	0.96 [0.95—0.99]	10
Light gradient boosting machine	0.89 [0.89—0.89]	0.80 [0.80—0.81]	0.81 [0.80—0.82]	0.46 [0.44—0.49]	0.95 [0.95—0.98]	15
Quadratic discriminant analysis	0.87 [0.87—0.89]	0.85 [0.84—0.89]	0.74 [0.73—0.75]	0.40 [0.40—0.41]	0.96 [0.95—0.99]	15

AUC, mean area under receiver operating curve. The numbers are presented as mean with 95%-confidence; PPV, positive predictive value; NPV, negative predictive value.

Table 2 shows the performance metrics for the top three ML models for prediction of 3-, 10-, 30- and 365-day mortality on test data based on the highest AUC and sensitivity performance. The best models were models with ten to fifteen variables. Performance metrics between training and test data were similar. The ML algorithms Naive Bayes, Linear Discriminant Analysis, and Logistic Regression had the highest mean AUC of 0.91–0.93 and sensitivity of 0.85–0.92, for 3-day mortality using 15 variables in the models (Table 2). For 10-day mortality, the ML algorithms Linear Discriminant Analysis and Quadratic Discriminant Analysis had the highest mean AUC of 0.90–0.91 and sensitivity of 0.90–91 using 10 variables. For 30-day mortality, the Linear Discriminant Analysis, Quadratic Discriminant Analysis, and Gradient Boosting Classifier had the highest mean AUC of 0.90–0.92 and sensitivity of 0.86–0.90 using 10 variables. Lastly, for 365-day mortality, the ML algorithms Gradient Boosting Classifier, the Light Gradient Boosting Machine, and Quadratic Discriminant Analysis had the highest mean AUC of 0.87–0.89 and a sensitivity of 0.80–0.85 using 10 to 15 variables (Table 2).

### Biomarker importance

**Figure 4:**
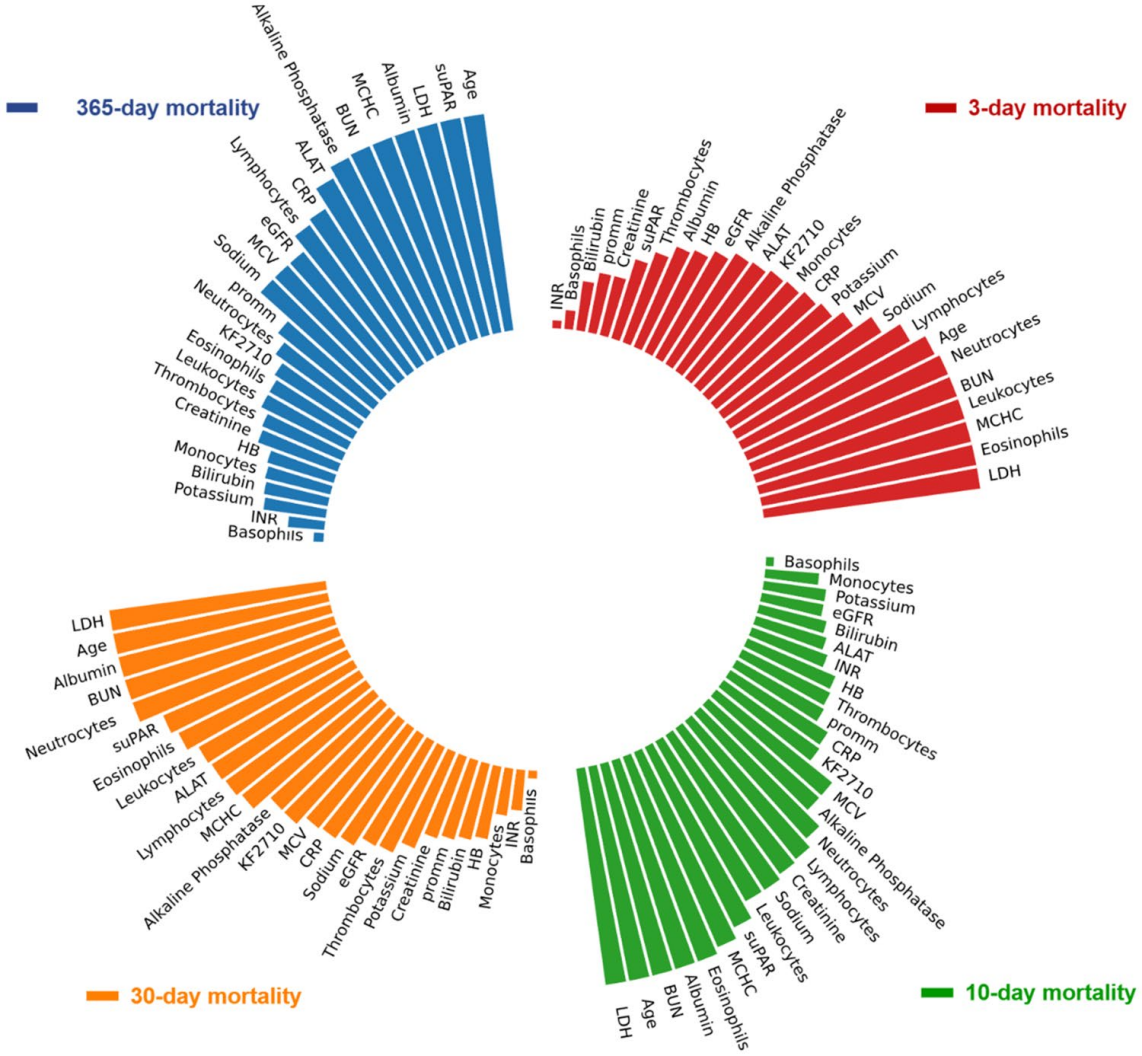
Ranking of importance of biomarkers in the IDA, LR, GBC, ADA and LightGBM models for prediction of 3-, 10, 30, and 365-day mortality.

Based on feature selection technique used on the IDA, LR, GBC, ADA and LightGBM models (ML algorithms in Methods), the biomarkers with the most importance for prediction of mortality were identified. Figure 4. Shows the top-ranked biomarkers for 3-, 10-, 30-, and 365-day mortality. Biomarkers like age, LDH, albumin, BUN, MCHC, are repeatedly ranked among the top variables in all models. Even when excluding age as a biomarker, the remaining variables where still top predictors and the predicted mortality for 3-, 10-, 30-, and 365-day remained showing very good performance AUC of > 0.80. Biomarkers like basophiles, INR, bilirubin, and monocytes are ranked in repeatedly among the lowest five in all models. Eosinophils, leukocytes, and neutrophils are among the biomarkers that move from top to bottom of the rank as follow-up time increases. In contrast, suPAR initially was ranked low at 3-day mortality outcome but rises to the top, at 365-day mortality, with an increase in follow-up time (Fig. 4).

## Discussion

The aim of this study was to develop and validate machine learning algorithms for finding high-mortality patients admitted to Emergency Departments using the results from routine blood testing and age. With as few as five biomarkers, machine learning-based algorithms provided very good performance predicting mortality in acutely admitted patients with AUC of 0.89 and 0.86, sensitivity of 0.83 and 0.80 for short and long-term mortality, respectively. Top three models, used between ten and fifteen biomarkers achieved an AUC of 90–93 and 87–89, sensitivity of 0.86–92 and 0.80–85 for short and long-term mortality, respectively. However, most models did not see an improvement from adding additional biomarkers. A similar study using few variables by Xie et al.^14^, developed and validated scores to predict the risk of death for ED patients using five to six biomarkers. These biomarkers included age, heart rate, respiration rate, diastolic blood pressure, systolic blood pressure, and cancer history. The 30-day score by Xie et al. achieved the best performance for mortality prediction, with an AUC of 0.82 (95%CI 0.81–0.83). However, similar to the very good discriminative performance of the scores, we further have demonstrated an excellent performance of > 0.90 by only using one routine blood sample. The models in our study are distinguished by their reliance on minimally modified data, primarily consisting of blood samples and patient age. This streamlined data approach stands in stark contrast to methods that require repetitive vital signs, medication profiles, and extensive disease histories, thereby significantly enhancing their applicability in clinical environments. Notably, these models align with current healthcare AI recommendations that advocate the use of high-quality, paraclinical data^51^. Such data is fundamental to the efficacy of AI applications in healthcare, providing robustness against stochastic errors. Furthermore, the simplicity of the data requirements in our models ensures scalability and adaptability across diverse clinical settings, a feature that is essential given the variety of cases encountered in emergency departments. This approach has the potential to democratize access to advanced diagnostic tools, broadening the use of machine learning and AI in clinical decision-making across a range of healthcare facilities. It's important to note that while our study focuses on the predictive capacity of blood tests, we acknowledge the critical prognostic implications of vital signs and the level of consciousness in emergency care. Our intention is not to sideline these factors but to explore additional, complementary diagnostic resources. Vital signs are crucial for assessing short-term or in-hospital mortality, but their utility in predicting long-term mortality is relatively limited^52–56^. In contrast, blood-based biomarkers can offer deeper insights into underlying pathologies, potentially improving the accuracy of long-term mortality predictions. This distinction becomes particularly crucial in the context of those cases where clinicians face uncertainty based solely on vital signs. These predictions can supplement the traditional reliance on vital signs, providing an additional layer of information that might tip the balance in clinical judgment for short- and long-term mortality outcomes.

In this study, we consider that clinically, it is easy to interpret and understand algorithms that can predict mortality based on the use of biomarkers, such as LDH, albumin, BUN, leukocyte and differential counts, and suPAR. An increase or decrease indicates underlying clinically pathological conditions which clinicians can comprehend, such as sever tissue damage, kidney disease and infection, and the levels of such biomarkers are stable overtime with only minor fluctuations. In contrast, abnormal values of vital signs as heart rate, respiration rate and blood pressure are either indicators of acute failure of the body’s most essential physiological functions, or an indication of compensatory physiological mechanisms in the heart or lungs and can fluctuate suddenly and significantly over minutes. Furthermore, clinically abnormal vital sign values need multiple recordings and re-evaluations ranging from four times per hour to two times per day to determine patients at risk of any deterioration.

### Biomarkers

Biomarker selection was essential since the practical use of algorithms with many clinical biomarkers are not feasible. All models ranked age, albumin, LDH, and BUN as key predictive factors. However, the ranks were different for short- and long-term mortality. In our study, the best predictors of short-term mortality are LDH, leukocyte counts and differential, BUN and MCHC while the best predictors of long-term mortality are age, LDH, suPAR, albumin and BUN. The biomarkers identified have previously been shown and used as prognostic and monitoring tools for diseases such as anemia, heart attack, bone fractures, muscle trauma, cancers, infections, inflammatory disorders, and hepatic-, renal-, and congestive heart failure^57–63^. These diseases are often found among frailty patients admitted to ED. Our results show that combining these biomarkers in one algorithm makes them valuable predictors for mortality.

### ML algorithms: new resources to find high-risk patients

This study's findings lay the groundwork for developing ML models that utilize a minimal set of biomarkers from a single blood sample. These models hold promise for future applications in identifying patients at risk following emergency admissions. Given the challenges posed by an aging population and crowded emergency departments globally, these tools could play a pivotal role in accurately determining patients' health statuses and directing resources efficiently towards high-risk individuals.

In various clinical scenarios, such mortality prediction algorithms can enhance patient safety and minimize preventable errors. These algorithms can serve as vital decision support tools, guiding actions such as hospitalization, discharge, delegation, and referral. For example, integrating short-term mortality prediction into the triage process in EDs, could assist to identify 'greyish patients'—those for whom clinicians face uncertainty regarding hospitalization decisions. This integration could be transformative in managing overcrowded EDs. Additionally, these models could be pivotal when assessing patients prior to discharge, particularly in identifying those at risk of mortality within the critical 10–30-day post-discharge period. Through the application of our ML algorithms to analyze a patient’s blood sample and relevant biomarkers, clinicians can extract valuable insights into a patient's risk profile. This information may not be evident through conventional clinical assessments alone. This predictive capability allows for a more nuanced discharge planning process, ensuring that patients who may require additional monitoring or follow-up care are appropriately identified. Such an approach not only enhances patient safety post-discharge but also aids in the effective allocation of post-hospitalization resources, potentially reducing readmission rates and improving overall patient outcomes.

Moreover, these algorithms have the potential to aid in complex decision-making scenarios, helping to avoid overtreatment and care that may not align with a patient's wishes and recovery capabilities. Understanding the appropriate level of medical intervention is a critical aspect of healthcare, particularly in scenarios where there is a mismatch between the care provided and patient preferences. In geriatric care, for example, it is often the case that aggressive interventions may be less beneficial for frail patients. Some may prefer less invasive treatments or prioritize quality of life over life prolongation. Respecting patient autonomy is a fundamental part of patient-centered care. By utilizing these predictive models as decision-making tools, clinicians and patients can engage in more informed and individualized healthcare decisions, ensuring an approach that aligns with patient preferences and improves their quality of life.

### Choosing the right machine learning model for mortality prediction

Our results reveal a crucial trend: the effectiveness of machine learning models varies based on the prediction timeframe. Simple models like logistic regression and Linear Discriminant Analysis are effective for short-term mortality (3–30 days), offering ease of use and interpretability, ideal for rapid clinical decisions. However, they may fall short in capturing complex, long-term data trends.

In contrast, advanced models like Light Gradient Boosting Machine and Gradient Boosting Classifier are effective in long-term mortality predictions, adept at analyzing complex data patterns. However, their complexity can be a double-edged sword; these models are often seen as `black boxes’ due to their lack of transparency, making it challenging for clinicians to understand the basis of their predictions.

The choice of the model, therefore requires careful consideration of these trade-offs. Clinicians and data scientists must consider both the prediction requirements and the need for model interpretability, ensuring the chosen model aligns with patient care's ethical and practical aspects.

## Conclusion

This study has demonstrated that high-risk of death in patients following admission can be identified by a routine blood sample, using a combination of five to fifteen biomarker measures. Eight of the fifteen evaluated ML algorithms achieved very good to excellent results of AUC (0.85–0.93). The ML algorithms Gradient Boosting Classifier, Light Gradient Boosting Machine, Linear Discriminant Analysis, Logistic regression, Naïve Bayes and Quadratic Discriminant Analysis showed the best performance on AUCs and sensitivity, even using only five biomarkers.

### Limitations and future research

To our knowledge, this is the first published study that has applied machine learning methods to predict acutely admitted emergency patients based on a few routine blood samples with excellent performance. There are, however, some limitations to this study. First, the data, which is over five years old, was retrospectively collected at a single clinical center. This raises concerns about the generalizability of the findings, as there may have been changes in methodologies, practices, population demographics, or environmental factors since the data was gathered. Second, our data encompassed patients from all specialties, except for children, gastroenterological, and obstetric patients. This exclusion of specific patient groups restricts the applicability of our trained models to these populations. Thirdly, our study did not integrate vital signs, due to lack of data and ethics approval, to perform a sensitivity analysis test. Fourthly, 4.3% of the total amount of patients with more than 50% missing data were excluded from the study, which could result in selection bias for the performance estimates. Fifthly, in this study we have used a probability threshold of 0.5, a more comprehensive analysis of the consequences of different thresholds is required to determine the right threshold. Last, but not least, machine learning techniques have also been criticized as black boxes by critics, so clinicians are skeptical of their use. This issue may be reduced by using interpretable biomarkers and using explaining ML tools or educating clinicians in ML concepts. Future work would need to focus on determining which algorithm should in the end be used, additional external validation would be needed to verify the robustness of this algorithm. The predictive performance of the ML models presented herein will be compared with existing warning scores for mortality prediction in follow-up validation studies. Implementation and prospective randomized trials would also be necessary to ensure the use and effectiveness of the algorithm.

### Supplementary Information

Supplementary Tables.

## Data Availability

The datasets analyzed during the current study are not publicly available due to privacy considerations (data use agreements) or ethical restrictions. However, they can be made available from the corresponding author upon reasonable request and after obtaining the necessary approvals from the relevant authorities.

## Code availability

The underlying code for this study, is not publicly available, for proprietary reasons. However, qualified researchers may request access from the corresponding author, and the code can be provided upon reasonable request. Access to the code used in this study can also be obtained through a private repository in GitHub.
