# Supplementary material for: Development and validation of prognostic machine learning models for short- and long-term mortality among acutely admitted patients based on blood tests
Source: Sci Rep. 2024 Mar 11;14:5942. doi: 10.1038/s41598-024-56638-6 (PMC10928126; doi:10.1038/s41598-024-56638-6)
Supplement: Supplementary file 1 — Supplementary Tables. [file 41598_2024_56638_MOESM1_ESM.pdf]

Supplementary tables

Supplementary Table 1: Missing Variables In The Dataset In Percent.

table 1:	
**Variables**	Missing in Percent %
Sex	0
Age	0
ALAT	9.9
Albumin	9.4
basophils	4.4
Alkaline Phosphatase	40
total Bilirubin	9.6
BUN	4.4
Creatinine	9.2
CRP	4.2
HB	3.3
INR	4.8
Potassium	3.6
KF2710	34.8
LDH	46.5
leukocytes	4.2
lymphocytes	4.4
MCHC	4.7
MCV	9.7
monocytes	4.4
neutrocytes	4.4
Promyelocytes	4.4
suPAR	11.9
thrombocytes	9.6
Eosinophils	44.4
eGFR	7.5
Sodium	3.2

Supplementary Table 2: Distribution of Missing Admission Data Variables: A Numerical and Percentage Analysis.

	Missing variable in numbers for admission data	Missing variables in percentage
**Number of admissions**		
**0.10 percentile**	0.0	0.0 %
**0.25 percentile**	1	2.6 %
**0.50 percentile**	1	2.6 %
**0.75 percentile**	3	7.7 %
**0.90 percentile**	6	15.4 %

Supplementary Table 3: Sensitivity Analysis: Model Performance including Comorbidities.

Model	AUC	Sensitivity	Specificity	PPV
**3-Day mortality**				
Naive Bayes	0.58 (0.54-0.62)	0.70 (0.61-0.78)	0.46 (0.46-0.47)	0.01 (0.01-0.01)
Logistic Regression	0.90 (0.86-0.93)	0.77 (0.68-0.84)	0.90 (0.90-0.91)	0.06 (0.05-0.07)
Linear Discriminant Analysis	0.89 (0.85-0.93)	0.80 (0.73-0.87)	0.89 (0.88-0.89)	0.05 (0.04-0.06)
**10- Day Mortality**				
Logistic Regression	0.92 (0.91-0.93)	0.82 (0.77-0.85)	0.86 (0.86-0.87)	0.14 (0.13-0.15)
Linear Discriminant Analysis	0.91 (0.90-0.93)	0.82 (0.78-0.87)	0.85 (0.84-0.85)	0.13 (0.12-0.14)
Quadratic Discriminant Analysis	0.50 (0.50-0.51)	0.01 (0.01-0.03)	0.99 (0.99-1.00)	0.07 (0.03-0.13)
**30-Day mortality**				
Linear Discriminant Analysis	0.91 (0.90-0.92)	0.83 (0.81-0.86)	0.82 (0.82-0.83)	0.21 (0.20-0.23)
Quadratic Discriminant Analysis	0.53 (0.52-0.54)	0.07 (0.05-0.09)	0.99 (0.99-0.99)	0.30 (0.23-0.37)
Gradient Boosting Classifier	0.91 (0.90-0.92)	0.84 (0.82-0.87)	0.83 (0.83-0.84)	0.23 (0.21-0.24)
**365-Day Mortality**				
Quadratic Discriminant Analysis	0.51 (0.51-0.51)	0.99 (0.99-0.99)	0.02 (0.02-0.03)	0.18 (0.17-0.19)
Light Gradient Boosting Machine	0.89 (0.89-0.90)	0.81 (0.79-0.82)	0.81 (0.80-0.82)	0.48 (0.46-0.49)
Gradient Boosting Classifier	0.88 (0.88-0.89)	0.82 (0.81-0.84)	0.78 (0.77-0.78)	0.44 (0.43-0.46)

These models were trained and tested including the biomarkers and comorbidities. Out of 4400 possible diagnoses, we selected 389 codes based on a prevalence threshold of over 50, ensuring that our analysis focused on the most prevalent and relevant comorbidities. The performance of these models was evaluated, with results including mean values and confidence interval.

Supplementary Table 4: Sensitivity Analysis: Model Performance Without Imputed Data.

Model	AUC	Sensitivity	Specificity	PPV	NPV
**3-Day mortality**					
Naive Bayes	0.86 (0.84-0.89)	0.81 (0.74-0.88)	0.81 (0.81-0.82)	0.03 (0.03-0.04)	1.00 (1.00-1.00)
Logistic Regression	0.90 (0.87-0.92)	0.85 (0.79-0.91)	0.80 (0.80-0.81)	0.03 (0.03-0.04)	1.00 (1.00-1.00)
Linear Discriminant Analysis	0.90 (0.88-0.93)	0.86 (0.80-0.92)	0.81 (0.80-0.82)	0.04 (0.03-0.04)	1.00 (1.00-1.00)
**10- Day Mortality**					
Logistic Regression	0.89 (0.88-0.90)	0.86 (0.83-0.89)	0.78 (0.77-0.79)	0.10 (0.09-0.11)	1.00 (0.99-1.00)
Linear Discriminant Analysis	0.89 (0.88-0.91)	0.87 (0.83-0.90)	0.78 (0.77-0.79)	0.10 (0.09-0.11)	1.00 (0.99-1.00)
Quadratic Discriminant Analysis	0.86 (0.84-0.87)	0.69 (0.64-0.74)	0.83 (0.83-0.84)	0.10 (0.09-0.12)	0.99 (0.99-0.99)
**30- Day Mortality**					
Linear Discriminant Analysis	0.89 (0.88-0.90)	0.85 (0.82-0.87)	0.78 (0.77-0.79)	0.19 (0.17-0.20)	0.99 (0.99-0.99)
Quadratic Discriminant Analysis	0.85 (0.83-0.85)	0.65 (0.62-0.68)	0.83 (0.83-0.84)	0.19 (0.18-0.20)	0.98 (0.97-0.98)
Gradient Boosting Classifier	0.90 (0.89-0.91)	0.86 (0.83-0.88)	0.79 (0.78-0.80)	0.19 (0.18-0.21)	0.99 (0.99-0.99)
**365-Day Mortality**					
Quadratic Discriminant Analysis	0.81 (0.80-0.82)	0.54 (0.52-0.56)	0.83 (0.83-0.84)	0.41 (0.39-0.42)	0.89 (0.89-0.90)
Light Gradient Boosting Machine	0.88 (0.87-0.88)	0.81 (0.80-0.83)	0.77 (0.76-0.78)	0.43 (0.42-0.44)	0.95 (0.95-0.96)
Gradient Boosting Classifier	0.87 (0.87-0.88)	0.84 (0.83-0.86)	0.74 (0.73-0.75)	0.41 (0.40-0.42)	0.96 (0.95-0.96)

Supplementary Table 5: Performance of All Models.

Variable	Model	Accuracy	AUC	Recall	Precision	Outcome
1	Ada Boost Classifier	0.7869	0.7442	0.6227	0.0215	3days
2	Ada Boost Classifier	0.6667	0.7464	0.7503	0.0131	3days
3	Ada Boost Classifier	0.8973	0.8605	0.6601	0.0337	3days
5	Ada Boost Classifier	0.9013	0.8842	0.6946	0.0321	3days
10	Ada Boost Classifier	0.9236	0.8863	0.6742	0.0353	3days
15	Ada Boost Classifier	0.9339	0.9005	0.7333	0.0421	3days
20	Ada Boost Classifier	0.9422	0.8948	0.6051	0.0400	3days

27	Ada Boost Classifier	0.9449	0.8763	0.6199	0.0415	3days
1	CatBoost Classifier	0.7553	0.6673	0.5313	0.0161	3days
2	CatBoost Classifier	0.9626	0.6628	0.0263	0.0046	3days
3	CatBoost Classifier	0.9866	0.7645	0.1405	0.0768	3days
5	CatBoost Classifier	0.9914	0.8317	0.0783	0.0738	3days
10	CatBoost Classifier	0.9939	0.8464	0.0995	0.1536	3days
15	CatBoost Classifier	0.9952	0.8612	0.1526	0.2858	3days
20	CatBoost Classifier	0.9952	0.8677	0.1449	0.2571	3days
27	CatBoost Classifier	0.9956	0.8922	0.1314	0.2947	3days
1	Decision Tree Classifier	0.8102	0.5021	0.2860	0.0113	3days
2	Decision Tree Classifier	0.9783	0.4973	0.0105	0.0035	3days
3	Decision Tree Classifier	0.9897	0.5178	0.0408	0.0496	3days
5	Decision Tree Classifier	0.9914	0.5080	0.0200	0.0327	3days
10	Decision Tree Classifier	0.9928	0.5365	0.0764	0.0801	3days
15	Decision Tree Classifier	0.9923	0.5377	0.0795	0.0735	3days
20	Decision Tree Classifier	0.9926	0.5337	0.0712	0.0794	3days
27	Decision Tree Classifier	0.9931	0.5188	0.0410	0.0442	3days
1	Extra Trees Classifier	0.8106	0.5014	0.2860	0.0114	3days
2	Extra Trees Classifier	0.9809	0.5080	0.0105	0.0045	3days
3	Extra Trees Classifier	0.9944	0.7534	0.0000	0.0000	3days
5	Extra Trees Classifier	0.9953	0.7956	0.0000	0.0000	3days
10	Extra Trees Classifier	0.9959	0.8393	0.0000	0.0000	3days
15	Extra Trees Classifier	0.9961	0.8555	0.0000	0.0000	3days
20	Extra Trees Classifier	0.9961	0.8398	0.0000	0.0000	3days
27	Extra Trees Classifier	0.9963	0.8726	0.0000	0.0000	3days
1	Extreme Gradient Boosting	0.8097	0.5868	0.3193	0.0126	3days
2	Extreme Gradient Boosting	0.9553	0.6791	0.0646	0.0096	3days
3	Extreme Gradient Boosting	0.9857	0.8155	0.1464	0.0715	3days
5	Extreme Gradient Boosting	0.9917	0.8437	0.0921	0.0925	3days
10	Extreme Gradient Boosting	0.9948	0.8698	0.1055	0.2368	3days
15	Extreme Gradient Boosting	0.9954	0.8677	0.1359	0.3486	3days
20	Extreme Gradient Boosting	0.9956	0.8785	0.0878	0.2669	3days
27	Extreme Gradient Boosting	0.9958	0.8872	0.1077	0.4210	3days
1	Gradient Boosting Classifier	0.7816	0.7398	0.5687	0.0192	3days
2	Gradient Boosting Classifier	0.7824	0.7477	0.6023	0.0158	3days
3	Gradient Boosting Classifier	0.9176	0.8653	0.6605	0.0415	3days
5	Gradient Boosting Classifier	0.9145	0.9014	0.6221	0.0335	3days
10	Gradient Boosting Classifier	0.9365	0.9125	0.6137	0.0384	3days
15	Gradient Boosting Classifier	0.9481	0.9123	0.6231	0.0459	3days
20	Gradient Boosting Classifier	0.9518	0.9075	0.5327	0.0424	3days
27	Gradient Boosting Classifier	0.9579	0.9209	0.5372	0.0471	3days
1	K Neighbors Classifier	0.7543	0.5262	0.3117	0.0096	3days
2	K Neighbors Classifier	0.9688	0.5192	0.0477	0.0106	3days

3	K Neighbors Classifier	0.9831	0.6273	0.1814	0.0722	3days
5	K Neighbors Classifier	0.9856	0.5944	0.1654	0.0699	3days
10	K Neighbors Classifier	0.9897	0.6311	0.2044	0.1058	3days
15	K Neighbors Classifier	0.9901	0.5586	0.1032	0.0568	3days
20	K Neighbors Classifier	0.9917	0.5589	0.0865	0.0659	3days
27	K Neighbors Classifier	0.9918	0.5457	0.0744	0.0545	3days
1	Light Gradient Boosting Machine	0.7525	0.7110	0.6183	0.0184	3days
2	Light Gradient Boosting Machine	0.9084	0.7185	0.1865	0.0123	3days
3	Light Gradient Boosting Machine	0.9692	0.8423	0.3399	0.0625	3days
5	Light Gradient Boosting Machine	0.9827	0.8763	0.2383	0.0745	3days
10	Light Gradient Boosting Machine	0.9920	0.8793	0.2286	0.1644	3days
15	Light Gradient Boosting Machine	0.9941	0.8953	0.1513	0.1977	3days
20	Light Gradient Boosting Machine	0.9950	0.8919	0.1513	0.3064	3days
27	Light Gradient Boosting Machine	0.9952	0.8995	0.1321	0.2620	3days
1	Linear Discriminant Analysis	0.7164	0.7677	0.6890	0.0179	3days
2	Linear Discriminant Analysis	0.6448	0.7586	0.7503	0.0121	3days
3	Linear Discriminant Analysis	0.7900	0.8737	0.7954	0.0197	3days
5	Linear Discriminant Analysis	0.8147	0.8946	0.8000	0.0198	3days
10	Linear Discriminant Analysis	0.8338	0.9226	0.8632	0.0207	3days
15	Linear Discriminant Analysis	0.8488	0.9286	0.8590	0.0218	3days
20	Linear Discriminant Analysis	0.8517	0.9231	0.8404	0.0217	3days
27	Linear Discriminant Analysis	0.8450	0.9244	0.8596	0.0203	3days
1	Logistic Regression	0.7105	0.7677	0.7013	0.0178	3days
2	Logistic Regression	0.6571	0.7594	0.7450	0.0124	3days
3	Logistic Regression	0.8001	0.8730	0.7895	0.0205	3days
5	Logistic Regression	0.8208	0.8944	0.7867	0.0201	3days
10	Logistic Regression	0.8472	0.9217	0.8330	0.0217	3days
15	Logistic Regression	0.8590	0.9266	0.8423	0.0229	3days
20	Logistic Regression	0.8597	0.9190	0.8327	0.0227	3days
27	Logistic Regression	0.8597	0.9201	0.8353	0.0218	3days
1	MLP Classifier	0.7702	0.7705	0.6393	0.0204	3days
2	MLP Classifier	0.7111	0.7737	0.7181	0.0143	3days
3	MLP Classifier	0.8770	0.8521	0.6788	0.0289	3days
5	MLP Classifier	0.9563	0.8513	0.4354	0.0485	3days
10	MLP Classifier	0.9898	0.8516	0.2143	0.1109	3days
15	MLP Classifier	0.9917	0.8250	0.1340	0.0927	3days
20	MLP Classifier	0.9933	0.8378	0.1192	0.1241	3days

27	MLP Classifier	0.9936	0.8736	0.0981	0.0976	3days
1	Naive Bayes	0.7605	0.7677	0.6642	0.0204	3days
2	Naive Bayes	0.6520	0.7389	0.6865	0.0113	3days
3	Naive Bayes	0.7612	0.8628	0.7833	0.0171	3days
5	Naive Bayes	0.7942	0.8867	0.8533	0.0189	3days
10	Naive Bayes	0.8035	0.9061	0.8698	0.0177	3days
15	Naive Bayes	0.7988	0.9113	0.9128	0.0175	3days
20	Naive Bayes	0.7855	0.8991	0.8705	0.0156	3days
27	Naive Bayes	0.8023	0.9091	0.8936	0.0166	3days
1	Quadratic Discriminant Analysis	0.7605	0.7677	0.6642	0.0204	3days
2	Quadratic Discriminant Analysis	0.6401	0.7986	0.8404	0.0134	3days
3	Quadratic Discriminant Analysis	0.7964	0.8800	0.8131	0.0207	3days
5	Quadratic Discriminant Analysis	0.8259	0.8999	0.8000	0.0210	3days
10	Quadratic Discriminant Analysis	0.8456	0.9046	0.8099	0.0209	3days
15	Quadratic Discriminant Analysis	0.8670	0.9121	0.7801	0.0226	3days
20	Quadratic Discriminant Analysis	0.8779	0.8910	0.7782	0.0243	3days
27	Quadratic Discriminant Analysis	0.8958	0.8912	0.7026	0.0249	3days
1	Random Forest Classifier	0.8102	0.5189	0.2860	0.0113	3days
2	Random Forest Classifier	0.9794	0.5387	0.0105	0.0038	3days
3	Random Forest Classifier	0.9938	0.7393	0.0176	0.1083	3days
5	Random Forest Classifier	0.9949	0.7805	0.0067	0.1000	3days
10	Random Forest Classifier	0.9959	0.8066	0.0302	0.3333	3days
15	Random Forest Classifier	0.9961	0.8269	0.0160	0.2000	3days
20	Random Forest Classifier	0.9961	0.8531	0.0000	0.0000	3days
27	Random Forest Classifier	0.9962	0.8611	0.0083	0.0500	3days
1	SVM - Radial Kernel	0.7716	0.7345	0.6477	0.0208	3days
2	SVM - Radial Kernel	0.7139	0.7950	0.7553	0.0151	3days
3	SVM - Radial Kernel	0.8475	0.8561	0.7310	0.0250	3days
5	SVM - Radial Kernel	0.8715	0.8554	0.6546	0.0233	3days
10	SVM - Radial Kernel	0.9570	0.8393	0.4495	0.0427	3days
15	SVM - Radial Kernel	0.9836	0.6991	0.2532	0.0661	3days
20	SVM - Radial Kernel	0.9915	0.6330	0.1981	0.1181	3days
27	SVM - Radial Kernel	0.9942	0.5879	0.0987	0.1459	3days
1	Ada Boost Classifier	0.7166	0.7280	0.5867	0.0058	10days
2	Ada Boost Classifier	0.8357	0.8712	0.7164	0.0904	10days
3	Ada Boost Classifier	0.8370	0.8885	0.7812	0.0951	10days
5	Ada Boost Classifier	0.8424	0.8933	0.7847	0.0856	10days

10	Ada Boost Classifier	0.8683	0.9053	0.7717	0.0971	10days
15	Ada Boost Classifier	0.8683	0.9079	0.8014	0.0987	10days
20	Ada Boost Classifier	0.8761	0.9138	0.7875	0.1016	10days
27	Ada Boost Classifier	0.8689	0.9055	0.7942	0.0952	10days
1	CatBoost Classifier	0.8003	0.6330	0.3589	0.0051	10days
2	CatBoost Classifier	0.8103	0.7448	0.4411	0.0965	10days
3	CatBoost Classifier	0.9253	0.8453	0.4536	0.1323	10days
5	CatBoost Classifier	0.9559	0.8549	0.3528	0.1671	10days
10	CatBoost Classifier	0.9706	0.8807	0.3026	0.2422	10days
15	CatBoost Classifier	0.9734	0.8847	0.2866	0.2626	10days
20	CatBoost Classifier	0.9763	0.8981	0.2946	0.3019	10days
27	CatBoost Classifier	0.9791	0.9028	0.2878	0.3580	10days
1	Decision Tree Classifier	0.8282	0.5414	0.2378	0.0040	10days
2	Decision Tree Classifier	0.9614	0.5463	0.1121	0.1095	10days
3	Decision Tree Classifier	0.9646	0.5580	0.1333	0.1435	10days
5	Decision Tree Classifier	0.9700	0.5674	0.1496	0.1594	10days
10	Decision Tree Classifier	0.9691	0.5732	0.1627	0.1531	10days
15	Decision Tree Classifier	0.9713	0.5764	0.1671	0.1718	10days
20	Decision Tree Classifier	0.9711	0.5678	0.1500	0.1541	10days
27	Decision Tree Classifier	0.9720	0.5767	0.1675	0.1696	10days
1	Extra Trees Classifier	0.8286	0.5468	0.2489	0.0041	10days
2	Extra Trees Classifier	0.9707	0.7158	0.0567	0.1205	10days
3	Extra Trees Classifier	0.9759	0.8132	0.0435	0.1997	10days
5	Extra Trees Classifier	0.9815	0.8497	0.0369	0.4750	10days
10	Extra Trees Classifier	0.9823	0.8887	0.0139	0.5500	10days
15	Extra Trees Classifier	0.9825	0.8956	0.0018	0.1000	10days
20	Extra Trees Classifier	0.9828	0.9132	0.0000	0.0000	10days
27	Extra Trees Classifier	0.9831	0.9010	0.0000	0.0000	10days
1	Extreme Gradient Boosting	0.8272	0.6354	0.2489	0.0041	10days
2	Extreme Gradient Boosting	0.9071	0.8378	0.4753	0.1126	10days
3	Extreme Gradient Boosting	0.9278	0.8565	0.4812	0.1436	10days
5	Extreme Gradient Boosting	0.9565	0.8560	0.3646	0.1733	10days
10	Extreme Gradient Boosting	0.9709	0.8794	0.2959	0.2423	10days
15	Extreme Gradient Boosting	0.9740	0.8800	0.2883	0.2744	10days
20	Extreme Gradient Boosting	0.9767	0.8877	0.2607	0.2959	10days
27	Extreme Gradient Boosting	0.9783	0.9006	0.2514	0.3199	10days
1	Gradient Boosting Classifier	0.7654	0.6925	0.4578	0.0055	10days
2	Gradient Boosting Classifier	0.8045	0.8705	0.7435	0.0783	10days
3	Gradient Boosting Classifier	0.8305	0.8934	0.7986	0.0932	10days
5	Gradient Boosting Classifier	0.8460	0.9006	0.7914	0.0882	10days
10	Gradient Boosting Classifier	0.8718	0.9192	0.7924	0.1018	10days
15	Gradient Boosting Classifier	0.8751	0.9210	0.7767	0.1012	10days
20	Gradient Boosting Classifier	0.8818	0.9241	0.7875	0.1060	10days

27	Gradient Boosting Classifier	0.8832	0.9175	0.7704	0.1036	10days
1	K Neighbors Classifier	0.8466	0.5415	0.2389	0.0044	10days
2	K Neighbors Classifier	0.9323	0.6588	0.2880	0.1071	10days
3	K Neighbors Classifier	0.9382	0.7038	0.3565	0.1363	10days
5	K Neighbors Classifier	0.9475	0.6913	0.3226	0.1286	10days
10	K Neighbors Classifier	0.9549	0.6805	0.2996	0.1392	10days
15	K Neighbors Classifier	0.9570	0.6679	0.2724	0.1371	10days
20	K Neighbors Classifier	0.9593	0.6679	0.2696	0.1417	10days
27	K Neighbors Classifier	0.9616	0.6483	0.2186	0.1275	10days
1	Light Gradient Boosting Machine	0.7976	0.6665	0.3578	0.0050	10days
2	Light Gradient Boosting Machine	0.8645	0.8570	0.6228	0.0959	10days
3	Light Gradient Boosting Machine	0.8834	0.8762	0.6594	0.1137	10days
5	Light Gradient Boosting Machine	0.9169	0.8865	0.5982	0.1269	10days
10	Light Gradient Boosting Machine	0.9388	0.9051	0.5484	0.1552	10days
15	Light Gradient Boosting Machine	0.9448	0.9081	0.5326	0.1653	10days
20	Light Gradient Boosting Machine	0.9553	0.9172	0.5268	0.1989	10days
27	Light Gradient Boosting Machine	0.9595	0.9134	0.4936	0.2079	10days
1	Linear Discriminant Analysis	0.6589	0.7188	0.7078	0.0058	10days
2	Linear Discriminant Analysis	0.7269	0.8498	0.8270	0.0625	10days
3	Linear Discriminant Analysis	0.7573	0.8834	0.8435	0.0697	10days
5	Linear Discriminant Analysis	0.7801	0.8908	0.8336	0.0659	10days
10	Linear Discriminant Analysis	0.8011	0.9103	0.8582	0.0722	10days
15	Linear Discriminant Analysis	0.8026	0.9088	0.8488	0.0710	10days
20	Linear Discriminant Analysis	0.8085	0.9120	0.8518	0.0722	10days
27	Linear Discriminant Analysis	0.8028	0.9066	0.8579	0.0693	10days
1	Logistic Regression	0.6695	0.7188	0.7078	0.0060	10days
2	Logistic Regression	0.7451	0.8493	0.8085	0.0654	10days
3	Logistic Regression	0.7792	0.8832	0.8159	0.0740	10days
5	Logistic Regression	0.7939	0.8906	0.8234	0.0693	10days
10	Logistic Regression	0.8186	0.9102	0.8530	0.0783	10days
15	Logistic Regression	0.8155	0.9087	0.8400	0.0750	10days
20	Logistic Regression	0.8217	0.9118	0.8464	0.0767	10days
27	Logistic Regression	0.8168	0.9064	0.8397	0.0730	10days
1	MLP Classifier	0.6588	0.7523	0.6644	0.0054	10days
2	MLP Classifier	0.7852	0.8746	0.7972	0.0761	10days
3	MLP Classifier	0.8197	0.8894	0.8000	0.0882	10days
5	MLP Classifier	0.8575	0.8755	0.7042	0.0865	10days

10	MLP Classifier	0.9421	0.8803	0.4880	0.1529	10days
15	MLP Classifier	0.9621	0.8648	0.3216	0.1806	10days
20	MLP Classifier	0.9663	0.8669	0.2411	0.1697	10days
27	MLP Classifier	0.9712	0.8544	0.2239	0.1972	10days
1	Naive Bayes	0.7219	0.7184	0.6522	0.0066	10days
2	Naive Bayes	0.7128	0.8581	0.8269	0.0595	10days
3	Naive Bayes	0.7436	0.8825	0.8551	0.0669	10days
5	Naive Bayes	0.7653	0.8876	0.8420	0.0625	10days
10	Naive Bayes	0.7704	0.8966	0.8720	0.0639	10days
15	Naive Bayes	0.7688	0.8926	0.8541	0.0614	10days
20	Naive Bayes	0.7825	0.9019	0.8768	0.0656	10days
27	Naive Bayes	0.7772	0.8955	0.8542	0.0615	10days
1	Quadratic Discriminant Analysis	0.7219	0.7184	0.6522	0.0066	10days
2	Quadratic Discriminant Analysis	0.6916	0.8662	0.8681	0.0581	10days
3	Quadratic Discriminant Analysis	0.7441	0.8895	0.8623	0.0675	10days
5	Quadratic Discriminant Analysis	0.7713	0.8901	0.8403	0.0639	10days
10	Quadratic Discriminant Analysis	0.8025	0.9100	0.8719	0.0737	10days
15	Quadratic Discriminant Analysis	0.7916	0.8977	0.8523	0.0677	10days
20	Quadratic Discriminant Analysis	0.8103	0.9061	0.8589	0.0733	10days
27	Quadratic Discriminant Analysis	0.8180	0.8999	0.8435	0.0737	10days
1	Random Forest Classifier	0.8282	0.5518	0.2378	0.0040	10days
2	Random Forest Classifier	0.9667	0.7525	0.1078	0.1450	10days
3	Random Forest Classifier	0.9730	0.8267	0.1072	0.2212	10days
5	Random Forest Classifier	0.9802	0.8485	0.1058	0.3773	10days
10	Random Forest Classifier	0.9815	0.8835	0.1021	0.4299	10days
15	Random Forest Classifier	0.9823	0.8975	0.0773	0.5460	10days
20	Random Forest Classifier	0.9827	0.8981	0.0714	0.4782	10days
27	Random Forest Classifier	0.9829	0.9033	0.0601	0.4673	10days
1	SVM - Radial Kernel	0.7160	0.7380	0.6422	0.0064	10days
2	SVM - Radial Kernel	0.7891	0.8555	0.7915	0.0769	10days
3	SVM - Radial Kernel	0.8078	0.8743	0.8348	0.0860	10days
5	SVM - Radial Kernel	0.8190	0.8805	0.7981	0.0762	10days
10	SVM - Radial Kernel	0.8821	0.8879	0.7162	0.1017	10days
15	SVM - Radial Kernel	0.9132	0.8860	0.6204	0.1193	10days
20	SVM - Radial Kernel	0.9446	0.8832	0.5000	0.1556	10days
27	SVM - Radial Kernel	0.9600	0.8381	0.4170	0.1904	10days
1	Ada Boost Classifier	0.8398	0.7908	0.6201	0.1505	30days

2	Ada Boost Classifier	0.8090	0.8707	0.7697	0.1485	30days
3	Ada Boost Classifier	0.8165	0.8885	0.8033	0.1625	30days
5	Ada Boost Classifier	0.8229	0.8918	0.7925	0.1691	30days
10	Ada Boost Classifier	0.8428	0.9045	0.8025	0.1845	30days
15	Ada Boost Classifier	0.8452	0.9058	0.8149	0.1795	30days
20	Ada Boost Classifier	0.8491	0.9093	0.8114	0.1847	30days
27	Ada Boost Classifier	0.8487	0.9082	0.8098	0.1878	30days
1	CatBoost Classifier	0.7695	0.8203	0.6956	0.1153	30days
2	CatBoost Classifier	0.8268	0.8501	0.6842	0.1491	30days
3	CatBoost Classifier	0.8733	0.8694	0.6387	0.1952	30days
5	CatBoost Classifier	0.8846	0.8692	0.6078	0.2105	30days
10	CatBoost Classifier	0.9315	0.8965	0.5654	0.3204	30days
15	CatBoost Classifier	0.9437	0.9069	0.5490	0.3622	30days
20	CatBoost Classifier	0.9465	0.9114	0.5442	0.3823	30days
27	CatBoost Classifier	0.9483	0.9161	0.5519	0.4045	30days
1	Decision Tree Classifier	0.7744	0.6576	0.4771	0.0872	30days
2	Decision Tree Classifier	0.8846	0.5789	0.2340	0.1027	30days
3	Decision Tree Classifier	0.9349	0.5922	0.2178	0.2230	30days
5	Decision Tree Classifier	0.9324	0.5836	0.2019	0.2079	30days
10	Decision Tree Classifier	0.9382	0.6129	0.2578	0.2623	30days
15	Decision Tree Classifier	0.9427	0.6131	0.2551	0.2665	30days
20	Decision Tree Classifier	0.9415	0.6194	0.2694	0.2703	30days
27	Decision Tree Classifier	0.9410	0.6202	0.2707	0.2756	30days
1	Extra Trees Classifier	0.7755	0.6786	0.4748	0.0872	30days
2	Extra Trees Classifier	0.8862	0.6763	0.2228	0.1005	30days
3	Extra Trees Classifier	0.9543	0.8367	0.1311	0.3799	30days
5	Extra Trees Classifier	0.9550	0.8527	0.0991	0.4175	30days
10	Extra Trees Classifier	0.9606	0.9038	0.0845	0.7719	30days
15	Extra Trees Classifier	0.9610	0.9107	0.0218	0.7033	30days
20	Extra Trees Classifier	0.9606	0.9101	0.0208	0.7750	30days
27	Extra Trees Classifier	0.9595	0.9142	0.0143	0.8000	30days
1	Extreme Gradient Boosting	0.7917	0.8171	0.6481	0.1202	30days
2	Extreme Gradient Boosting	0.8402	0.8568	0.6654	0.1576	30days
3	Extreme Gradient Boosting	0.8763	0.8751	0.6343	0.1988	30days
5	Extreme Gradient Boosting	0.8905	0.8687	0.5713	0.2135	30days
10	Extreme Gradient Boosting	0.9294	0.8896	0.5397	0.3067	30days
15	Extreme Gradient Boosting	0.9421	0.9037	0.4992	0.3421	30days
20	Extreme Gradient Boosting	0.9440	0.8994	0.4788	0.3535	30days
27	Extreme Gradient Boosting	0.9497	0.9083	0.4880	0.4059	30days
1	Gradient Boosting Classifier	0.8197	0.8124	0.6503	0.1382	30days
2	Gradient Boosting Classifier	0.7933	0.8741	0.8079	0.1431	30days
3	Gradient Boosting Classifier	0.8065	0.8919	0.8251	0.1578	30days
5	Gradient Boosting Classifier	0.8033	0.8941	0.8274	0.1589	30days

10	Gradient Boosting Classifier	0.8419	0.9145	0.8341	0.1882	30days
15	Gradient Boosting Classifier	0.8460	0.9197	0.8274	0.1822	30days
20	Gradient Boosting Classifier	0.8510	0.9206	0.8260	0.1888	30days
27	Gradient Boosting Classifier	0.8536	0.9234	0.8301	0.1962	30days
1	K Neighbors Classifier	0.7518	0.7383	0.5863	0.0939	30days
2	K Neighbors Classifier	0.8539	0.6954	0.4245	0.1248	30days
3	K Neighbors Classifier	0.8940	0.7379	0.4690	0.1919	30days
5	K Neighbors Classifier	0.8927	0.7366	0.4566	0.1900	30days
10	K Neighbors Classifier	0.9130	0.7604	0.4993	0.2407	30days
15	K Neighbors Classifier	0.9174	0.7451	0.4378	0.2237	30days
20	K Neighbors Classifier	0.9222	0.7360	0.4311	0.2393	30days
27	K Neighbors Classifier	0.9225	0.7433	0.4391	0.2493	30days
1	Light Gradient Boosting Machine	0.7738	0.8215	0.6873	0.1164	30days
2	Light Gradient Boosting Machine	0.8098	0.8653	0.7622	0.1478	30days
3	Light Gradient Boosting Machine	0.8408	0.8851	0.7480	0.1758	30days
5	Light Gradient Boosting Machine	0.8469	0.8842	0.7276	0.1818	30days
10	Light Gradient Boosting Machine	0.8953	0.9073	0.7159	0.2449	30days
15	Light Gradient Boosting Machine	0.9066	0.9184	0.7154	0.2563	30days
20	Light Gradient Boosting Machine	0.9117	0.9214	0.7098	0.2705	30days
27	Light Gradient Boosting Machine	0.9176	0.9241	0.7226	0.2949	30days
1	Linear Discriminant Analysis	0.6836	0.7607	0.7392	0.0900	30days
2	Linear Discriminant Analysis	0.7522	0.8490	0.7945	0.1199	30days
3	Linear Discriminant Analysis	0.7416	0.8680	0.8362	0.1233	30days
5	Linear Discriminant Analysis	0.7607	0.8754	0.8374	0.1346	30days
10	Linear Discriminant Analysis	0.7918	0.8971	0.8561	0.1509	30days
15	Linear Discriminant Analysis	0.7924	0.9011	0.8483	0.1428	30days
20	Linear Discriminant Analysis	0.8005	0.9032	0.8506	0.1497	30days
27	Linear Discriminant Analysis	0.8002	0.9038	0.8609	0.1538	30days
1	Logistic Regression	0.6996	0.7607	0.7279	0.0933	30days
2	Logistic Regression	0.7616	0.8490	0.7825	0.1228	30days
3	Logistic Regression	0.7617	0.8679	0.8158	0.1302	30days
5	Logistic Regression	0.7760	0.8754	0.8246	0.1413	30days
10	Logistic Regression	0.8046	0.8973	0.8436	0.1579	30days
15	Logistic Regression	0.8075	0.9013	0.8352	0.1510	30days
20	Logistic Regression	0.8124	0.9035	0.8368	0.1561	30days
27	Logistic Regression	0.8125	0.9042	0.8466	0.1608	30days
1	MLP Classifier	0.8182	0.7897	0.6548	0.1378	30days

2	MLP Classifier	0.7940	0.8773	0.8185	0.1448	30days
3	MLP Classifier	0.8054	0.8911	0.8289	0.1578	30days
5	MLP Classifier	0.8122	0.8873	0.8096	0.1631	30days
10	MLP Classifier	0.8866	0.8764	0.6483	0.2171	30days
15	MLP Classifier	0.9232	0.8809	0.5615	0.2725	30days
20	MLP Classifier	0.9358	0.8601	0.4296	0.2929	30days
27	MLP Classifier	0.9395	0.8671	0.4000	0.3135	30days
1	Naive Bayes	0.7347	0.7606	0.7158	0.1036	30days
2	Naive Bayes	0.7504	0.8558	0.8065	0.1204	30days
3	Naive Bayes	0.7225	0.8714	0.8609	0.1182	30days
5	Naive Bayes	0.7411	0.8737	0.8445	0.1263	30days
10	Naive Bayes	0.7752	0.8876	0.8554	0.1409	30days
15	Naive Bayes	0.7709	0.8896	0.8561	0.1318	30days
20	Naive Bayes	0.7758	0.8932	0.8660	0.1368	30days
27	Naive Bayes	0.7742	0.8912	0.8632	0.1384	30days
1	Quadratic Discriminant Analysis	0.7347	0.7606	0.7158	0.1036	30days
2	Quadratic Discriminant Analysis	0.7440	0.8632	0.8320	0.1206	30days
3	Quadratic Discriminant Analysis	0.7258	0.8799	0.8762	0.1211	30days
5	Quadratic Discriminant Analysis	0.7472	0.8817	0.8659	0.1314	30days
10	Quadratic Discriminant Analysis	0.7808	0.8952	0.8745	0.1464	30days
15	Quadratic Discriminant Analysis	0.7816	0.8927	0.8639	0.1384	30days
20	Quadratic Discriminant Analysis	0.7917	0.8969	0.8584	0.1450	30days
27	Quadratic Discriminant Analysis	0.7978	0.8942	0.8474	0.1506	30days
1	Random Forest Classifier	0.7747	0.7144	0.4778	0.0874	30days
2	Random Forest Classifier	0.8823	0.7406	0.2520	0.1063	30days
3	Random Forest Classifier	0.9474	0.8372	0.2185	0.3190	30days
5	Random Forest Classifier	0.9501	0.8495	0.1969	0.3573	30days
10	Random Forest Classifier	0.9598	0.9003	0.2504	0.5448	30days
15	Random Forest Classifier	0.9625	0.9136	0.1827	0.5837	30days
20	Random Forest Classifier	0.9618	0.9155	0.1901	0.5652	30days
27	Random Forest Classifier	0.9622	0.9177	0.2211	0.6004	30days
1	SVM - Radial Kernel	0.8292	0.7740	0.6428	0.1442	30days
2	SVM - Radial Kernel	0.7822	0.8533	0.8289	0.1390	30days
3	SVM - Radial Kernel	0.7903	0.8702	0.8434	0.1494	30days
5	SVM - Radial Kernel	0.7897	0.8734	0.8545	0.1532	30days
10	SVM - Radial Kernel	0.8469	0.8930	0.7981	0.1879	30days
15	SVM - Radial Kernel	0.8800	0.8987	0.7325	0.2098	30days

20	SVM - Radial Kernel	0.9075	0.9000	0.6713	0.2530	30days
27	SVM - Radial Kernel	0.9166	0.9037	0.6301	0.2744	30days
1	Ada Boost Classifier	0.6200	0.7526	0.7991	0.2877	365days
2	Ada Boost Classifier	0.7321	0.8255	0.7702	0.3631	365days
3	Ada Boost Classifier	0.7485	0.8389	0.7843	0.3772	365days
5	Ada Boost Classifier	0.7712	0.8589	0.7879	0.3936	365days
10	Ada Boost Classifier	0.7851	0.8704	0.7931	0.4071	365days
15	Ada Boost Classifier	0.7912	0.8765	0.8021	0.4123	365days
20	Ada Boost Classifier	0.7903	0.8762	0.8012	0.4088	365days
27	Ada Boost Classifier	0.7914	0.8777	0.7983	0.4125	365days
1	CatBoost Classifier	0.6273	0.7517	0.7890	0.2905	365days
2	CatBoost Classifier	0.7336	0.8225	0.7582	0.3629	365days
3	CatBoost Classifier	0.7567	0.8359	0.7621	0.3836	365days
5	CatBoost Classifier	0.7922	0.8593	0.7568	0.4185	365days
10	CatBoost Classifier	0.8212	0.8793	0.7462	0.4591	365days
15	CatBoost Classifier	0.8336	0.8881	0.7475	0.4780	365days
20	CatBoost Classifier	0.8379	0.8897	0.7374	0.4839	365days
27	CatBoost Classifier	0.8443	0.8957	0.7387	0.5000	365days
1	Decision Tree Classifier	0.6271	0.7409	0.7588	0.2856	365days
2	Decision Tree Classifier	0.7288	0.6657	0.5140	0.3154	365days
3	Decision Tree Classifier	0.7931	0.6253	0.3734	0.3774	365days
5	Decision Tree Classifier	0.8097	0.6445	0.4014	0.4055	365days
10	Decision Tree Classifier	0.8202	0.6611	0.4287	0.4301	365days
15	Decision Tree Classifier	0.8240	0.6612	0.4249	0.4330	365days
20	Decision Tree Classifier	0.8261	0.6601	0.4198	0.4350	365days
27	Decision Tree Classifier	0.8255	0.6588	0.4167	0.4368	365days
1	Extra Trees Classifier	0.6271	0.7409	0.7588	0.2856	365days
2	Extra Trees Classifier	0.7354	0.6855	0.5129	0.3230	365days
3	Extra Trees Classifier	0.8243	0.7878	0.3367	0.4621	365days
5	Extra Trees Classifier	0.8597	0.8544	0.3302	0.6176	365days
10	Extra Trees Classifier	0.8745	0.8822	0.3268	0.7287	365days
15	Extra Trees Classifier	0.8740	0.8868	0.2852	0.7512	365days
20	Extra Trees Classifier	0.8732	0.8885	0.2640	0.7575	365days
27	Extra Trees Classifier	0.8743	0.8924	0.2646	0.7861	365days
1	Extreme Gradient Boosting	0.6269	0.7480	0.7774	0.2885	365days
2	Extreme Gradient Boosting	0.7342	0.8192	0.7531	0.3629	365days
3	Extreme Gradient Boosting	0.7572	0.8317	0.7455	0.3823	365days
5	Extreme Gradient Boosting	0.7951	0.8528	0.7245	0.4199	365days
10	Extreme Gradient Boosting	0.8231	0.8702	0.7093	0.4607	365days
15	Extreme Gradient Boosting	0.8330	0.8795	0.7049	0.4757	365days
20	Extreme Gradient Boosting	0.8386	0.8789	0.6924	0.4845	365days
27	Extreme Gradient Boosting	0.8452	0.8840	0.6879	0.5021	365days
1	Gradient Boosting Classifier	0.6193	0.7543	0.7995	0.2873	365days

2	Gradient Boosting Classifier	0.7287	0.8276	0.7789	0.3606	365days
3	Gradient Boosting Classifier	0.7451	0.8426	0.7975	0.3750	365days
5	Gradient Boosting Classifier	0.7692	0.8639	0.8032	0.3928	365days
10	Gradient Boosting Classifier	0.7863	0.8792	0.8169	0.4108	365days
15	Gradient Boosting Classifier	0.7910	0.8846	0.8203	0.4136	365days
20	Gradient Boosting Classifier	0.7912	0.8852	0.8236	0.4122	365days
27	Gradient Boosting Classifier	0.7959	0.8882	0.8210	0.4205	365days
1	K Neighbors Classifier	0.6334	0.6898	0.6534	0.2711	365days
2	K Neighbors Classifier	0.7069	0.7331	0.6422	0.3188	365days
3	K Neighbors Classifier	0.7233	0.7377	0.6499	0.3312	365days
5	K Neighbors Classifier	0.7528	0.7697	0.6756	0.3570	365days
10	K Neighbors Classifier	0.7799	0.7936	0.6988	0.3897	365days
15	K Neighbors Classifier	0.7895	0.8053	0.7039	0.3998	365days
20	K Neighbors Classifier	0.7969	0.8039	0.6976	0.4080	365days
27	K Neighbors Classifier	0.7991	0.8128	0.7005	0.4143	365days
1	Light Gradient Boosting Machine	0.6289	0.7508	0.7774	0.2897	365days
2	Light Gradient Boosting Machine	0.7354	0.8255	0.7673	0.3660	365days
3	Light Gradient Boosting Machine	0.7530	0.8392	0.7777	0.3813	365days
5	Light Gradient Boosting Machine	0.7802	0.8623	0.7852	0.4047	365days
10	Light Gradient Boosting Machine	0.8024	0.8805	0.7898	0.4311	365days
15	Light Gradient Boosting Machine	0.8098	0.8880	0.7890	0.4383	365days
20	Light Gradient Boosting Machine	0.8134	0.8885	0.7911	0.4420	365days
27	Light Gradient Boosting Machine	0.8198	0.8927	0.7903	0.4549	365days
1	Linear Discriminant Analysis	0.6607	0.7537	0.7272	0.3029	365days
2	Linear Discriminant Analysis	0.7169	0.8241	0.7958	0.3517	365days
3	Linear Discriminant Analysis	0.7291	0.8407	0.8214	0.3617	365days
5	Linear Discriminant Analysis	0.7456	0.8537	0.8188	0.3683	365days
10	Linear Discriminant Analysis	0.7641	0.8673	0.8294	0.3851	365days
15	Linear Discriminant Analysis	0.7670	0.8718	0.8338	0.3851	365days
20	Linear Discriminant Analysis	0.7672	0.8728	0.8317	0.3832	365days
27	Linear Discriminant Analysis	0.7706	0.8753	0.8354	0.3896	365days
1	Logistic Regression	0.6664	0.7537	0.7175	0.3055	365days
2	Logistic Regression	0.7295	0.8241	0.7795	0.3616	365days
3	Logistic Regression	0.7415	0.8406	0.8023	0.3718	365days
5	Logistic Regression	0.7565	0.8537	0.8050	0.3785	365days
10	Logistic Regression	0.7743	0.8674	0.8132	0.3953	365days
15	Logistic Regression	0.7770	0.8720	0.8169	0.3953	365days

20	Logistic Regression	0.7777	0.8730	0.8196	0.3944	365days
27	Logistic Regression	0.7812	0.8756	0.8224	0.4011	365days
1	MLP Classifier	0.6248	0.7537	0.7889	0.2889	365days
2	MLP Classifier	0.7278	0.8273	0.7800	0.3600	365days
3	MLP Classifier	0.7375	0.8427	0.8104	0.3686	365days
5	MLP Classifier	0.7701	0.8621	0.7973	0.3934	365days
10	MLP Classifier	0.7976	0.8713	0.7802	0.4241	365days
15	MLP Classifier	0.8129	0.8676	0.7316	0.4398	365days
20	MLP Classifier	0.8149	0.8600	0.7025	0.4388	365days
27	MLP Classifier	0.8232	0.8552	0.6638	0.4540	365days
1	Naive Bayes	0.6286	0.7537	0.7860	0.2908	365days
2	Naive Bayes	0.7050	0.8237	0.8056	0.3424	365days
3	Naive Bayes	0.7221	0.8395	0.8210	0.3550	365days
5	Naive Bayes	0.7417	0.8528	0.8222	0.3648	365days
10	Naive Bayes	0.7493	0.8549	0.8138	0.3671	365days
15	Naive Bayes	0.7577	0.8593	0.8167	0.3729	365days
20	Naive Bayes	0.7599	0.8581	0.8108	0.3727	365days
27	Naive Bayes	0.7639	0.8594	0.8121	0.3795	365days
1	Quadratic Discriminant Analysis	0.6286	0.7537	0.7860	0.2908	365days
2	Quadratic Discriminant Analysis	0.6954	0.8253	0.8213	0.3365	365days
3	Quadratic Discriminant Analysis	0.7104	0.8387	0.8399	0.3470	365days
5	Quadratic Discriminant Analysis	0.7316	0.8551	0.8451	0.3576	365days
10	Quadratic Discriminant Analysis	0.7540	0.8689	0.8444	0.3756	365days
15	Quadratic Discriminant Analysis	0.7605	0.8685	0.8383	0.3783	365days
20	Quadratic Discriminant Analysis	0.7613	0.8624	0.8176	0.3751	365days
27	Quadratic Discriminant Analysis	0.7655	0.8607	0.8079	0.3809	365days
1	Random Forest Classifier	0.6239	0.7408	0.7629	0.2843	365days
2	Random Forest Classifier	0.7277	0.7396	0.5344	0.3187	365days
3	Random Forest Classifier	0.8092	0.7974	0.4422	0.4297	365days
5	Random Forest Classifier	0.8517	0.8523	0.4644	0.5442	365days
10	Random Forest Classifier	0.8700	0.8800	0.4759	0.6138	365days
15	Random Forest Classifier	0.8749	0.8884	0.4629	0.6349	365days
20	Random Forest Classifier	0.8752	0.8894	0.4591	0.6327	365days
27	Random Forest Classifier	0.8783	0.8931	0.4600	0.6565	365days
1	SVM - Radial Kernel	0.6237	0.7335	0.7922	0.2888	365days
2	SVM - Radial Kernel	0.7220	0.8063	0.7889	0.3555	365days
3	SVM - Radial Kernel	0.7339	0.8176	0.8178	0.3659	365days

5	SVM - Radial Kernel	0.7571	0.8439	0.8232	0.3810	365days
10	SVM - Radial Kernel	0.7791	0.8687	0.8313	0.4030	365days
15	SVM - Radial Kernel	0.7989	0.8769	0.8120	0.4238	365days
20	SVM - Radial Kernel	0.8043	0.8799	0.8036	0.4289	365days
27	SVM - Radial Kernel	0.8190	0.8864	0.7870	0.4533	365days
